# Supplementary material for: Content-rich biological network constructed by mining PubMed abstracts
Source: BMC Bioinformatics. 2004 Oct 8;5:147. doi: 10.1186/1471-2105-5-147 (PMC528731; doi:10.1186/1471-2105-5-147)
Supplement: Additional File 2 — The original results of the above study (non-essential files are deleted to keep the file size under the limit set by BMC bioinformatics). [file 1471-2105-5-147-S2.bz2 › chilibotAdditionalFile2/dip10/598ID9799632E10977/html/SINR_SINI.html]

 


 **SINR** and **SINI** 
  
Found 8 abstracts in PubMed, retrieved 8.  
 

 What does Google say? 
 PDF only 
| .edu only 

---

**Interactive relationship** (e.g. stimulation, inhibition, etc)

**Neutral relationship**- The interactions governing this curious quaternary transition are revealed in the crystal structure of the  **SinI**   **SinR**  complex.  Ref: 9799632 J Mol Biol, 1998
- Thus,  **SinI**  acts as an antagonist to  **SinR** .  Ref: 8422983 Genes Dev, 1993
- **SinI**  modulates the activity of  **SinR** , a developmental switch protein of Bacillus subtilis, by protein protein interaction.  Ref: 8422983 Genes Dev, 1993
- This repression is overcome through the activity of  **SinI** , which disrupts the  **SinR**  tetramer through the formation of a  **SinI**   **SinR**  heterodimer.  Ref: 9799632 J Mol Biol, 1998
- A chromosomal in frame deletion of  **sinI**  gives rise to a phenotype associated with higher levels of  **SinR** .  Ref: 8422983 Genes Dev, 1993
- We demonstrate that the oligomeric forms of  **SinR**  and  **SinI**  re arrange to form a tight 1 1  **SinR**   **SinI**  complex, with no stable intermediate species.  Ref: 10547280 J Mol Biol, 1999
- In vivo experiments have demonstrated that overexpression of  **sinI**  results in phenotypes that are observed in cells with a null mutation of  **sinR** .  Ref: 8422983 Genes Dev, 1993
- We show in this discussion that the product of  **sinI** , the first open reading frame ORF of this operon, interferes with the function of  **SinR** .  Ref: 8422983 Genes Dev, 1993
- The transcription factor  **SinR** , a pleiotropic regulator of late growth processes in Bacillus subtilis, has been crystallised as a complex with its antagonist  **SinI** , in a form suitable for structural analysis.  Ref: 8549812 FEBS Lett, 1996
- **SinR**  is a DNA binding protein regulating gene expression and  **SinI**  inhibits  **SinR**  activity.  Ref: 11429458 Microbiology, 2001
- Crystallisation of the Bacillus subtilis sporulation inhibitor  **SinR** , complexed with its antagonist,  **SinI** .  Ref: 8549812 FEBS Lett, 1996

**Non-interactive relationship** (e.g. studied together, co-existance, homology, etc.)

- The protein protein interaction between  **SinI**  and  **SinR**  has been studied by analytical ultracentrifugation and gel electrophoresis in an attempt to understand how these proteins contribute to developmental control of sporulation in Bacillus subtilis.  Ref: 10547280 J Mol Biol, 1999
- Recent work has identified a pair of genes,  **sinR**  and  **sinI** , which represent a potential quorum sensing system and are responsible for the production of N acyl homoserine lactones AHLs in two S. meliloti strains, Rm1021 and Rm41.  Ref: 12270827 J Bacteriol, 2002
- **SinI**  prevents  **SinR**  from binding to its target sequence on aprE, and the two proteins form a complex that can be immunoprecipitated with antibodies to either  **SinR**  or  **SinI** .  Ref: 8422983 Genes Dev, 1993
- The  **SinI**   **SinR**  crystals diffract X rays generated from a rotating copper anode source to 2.3 A spacing and a complete native dataset has been collected to this resolution limit.  Ref: 8549812 FEBS Lett, 1996
- **SinR**  was found to be tetrameric, while  **SinI**  was found to exist as monomers and dimers in a rapidly reversible equilibrium.  Ref: 10547280 J Mol Biol, 1999
- Changes to stage II gene expression could be linked to alterations in the expression of the  **sinI**  and  **sinR**  genes.  Ref: 11466285 J Bacteriol, 2001
- We also show that  **sinI**  and  **sinR**  mutants are still capable of producing several short chain AHLs, one of which was identified as octanoyl HL.  Ref: 12270827 J Bacteriol, 2002
- We also present evidence for the existence of a second quorum sensing system,  **sinR**   **sinI** , which is present in both AK631 and Rm1021.  Ref: 12057940 J Bacteriol, 2002
- Labelling of  **SinR**  by incorporating the tryptophan analogue 7 azatryptophan 7AW into the protein in place of tryptophan shifts the UV absorbance spectrum, thus allowing selective monitoring of 7AWSinR at 315 nm using the UV absorption optics of the analytical ultracentrifuge.Selective monitoring of  **SinR**  in mixtures of  **SinR**  and  **SinI**  enables the binding and stoichiometry of the interaction to be investigated quantitatively and unambiguously.  Ref: 10547280 J Mol Biol, 1999
- Moreover, the same  **sinI**  and  **sinR**  mutations also lead to a decrease in the number of pink nodules during nodulation assays, as well as a slight delay in the appearance of pink nodules, indicating a role for quorum sensing in symbiosis.  Ref: 12270827 J Bacteriol, 2002
- Assuming that there is a single  **SinI**   **SinR**  heterodimer in the asymmetric unit, the crystals have a Vm of 2.53 A3.Da 1.  Ref: 8549812 FEBS Lett, 1996
- Characterization of the Sinorhizobium meliloti  **sinR**   **sinI**  locus and the production of novel N acyl homoserine lactones.  Ref: 12270827 J Bacteriol, 2002
- The DNA region upstream from inhA contains two genes encoding polypeptides similar to the  **SinI**  and  **SinR**  regulators of B. subtilis.  Ref: 11429458 Microbiology, 2001
